# Genomic copy number alterations as biomarkers for triple negative pregnancy-associated breast cancer

**DOI:** 10.1007/s13402-022-00685-6

**Published:** 2022-07-06

**Authors:** B. B. M. Suelmann, A. Rademaker, C. van Dooijeweert, E. van der Wall, P. J. van Diest, C. B. Moelans

**Affiliations:** 1 Department of Medical Oncology, University Medical Center Utrecht, Utrecht, The Netherlands; 2 Department of Pathology, University Medical Center Utrecht, PO Box 85500, Utrecht, 3508 GA The Netherlands

**Keywords:** Breast cancer, Pregnancy, Biomarkers, MLPA, Copy number alteration

## Abstract

**Background:**

PABC, commonly defined as breast cancer diagnosed during or ≤ 1 year after pregnancy, accounts for 7% of all breast cancers in women ≤ 45 years. Compared to age-matched non-PABC patients, PABC is characterized by a particularly aggressive histopathologic profile with poorly differentiated and estrogen- and progesterone receptor negative tumors and associated high mortality rates. This study assessed the genomic background of triple-negative PABC tumors by detection of copy number alterations (CNAs).

**Methods:**

MLPA was used to compare CNAs in breast cancer-associated chromosomal loci between triple-negative PABC- and subtype-matched non-PABC patients. Both CNA patterns were evaluated by cluster analysis; associations between individual gene CNAs, pathological characteristics and survival were explored.

**Results:**

Triple-negative PABC tumors exhibited unique CNAs compared to non-PABC tumors, including enrichment for *TOP2A* copy number loss, an independent predictor of worse overall survival (HR 8.96, *p* = 0.020). Cluster analysis based on CNA profiles identified a triple-negative PABC-subgroup with a particularly poor prognosis, characterized by chromosome 8p copy number loss. Individual gene CNAs analysis revealed that *FGFR1* copy number loss on chromosome 8p11.23 was an independent predictor of poor outcome in multivariate analysis (HR 3.59, *p* = 0.053) and predicted the development of distant metastases (*p* = 0.048).

**Conclusion:**

This study provides novel insights into the biology of triple-negative PABC tumors suggesting that CNAs, particularly 8p loss and *TOP2A* loss, are involved in the development of breast cancer during pregnancy. *FGFR1* loss and *TOP2A* loss seem to be promising new biomarkers that independently identify subgroups of PABC patients with poor prognosis. These genomic biomarkers may provide clues for personalized therapy.

**Supplementary Information:**

The online version contains supplementary material available at 10.1007/s13402-022-00685-6.

## Introduction

Breast cancer is the most common malignancy diagnosed during pregnancy and its incidence is rising notably due to the present-day trend of delayed childbearing, the increase of young-onset breast cancer and the introduction of non-invasive prenatal testing (NIPT) in obstetrical care (resulting in the accidental identification of several asymptomatic pregnant patients in developed countries) [1–4].

Pregnancy-associated breast cancer (PABC), generally defined as breast cancer diagnosed anytime during gestation, lactation or within one year after delivery, represents a heterogeneous disease with fundamental histological and clinical variation between patients. Every year, one in 3,000 to 10,000 pregnant women are diagnosed with breast cancer, representing only 0.2 – 3.8% of overall breast cancer cases [2, 5–7].

The molecular nature of PABC remains an underexplored field, and considerable controversy exists regarding the influence of pregnancy on breast cancer prognosis [8–16]. PABC is generally believed to exhibit particularly aggressive behavior and its poor outcome is largely attributed to unfavorable tumor characteristics: advanced tumor (T) stage at diagnosis, lymph node involvement, high histologic grade, negative estrogen receptor (ER) and progesterone receptor (PR) status, and human epidermal growth factor receptor-2 (HER-2) amplification and overexpression [13, 14, 17, 18]. To date, little progress has been made in unraveling the molecular mechanisms of the aggressive pathological characteristics of PABC tumors. A deeper understanding of the molecular makeup of PABC may not only help explain its aggressive biological attributes, but may also provide individualized biomarkers and potential targets for new cancer therapies.

Somatic copy number alterations (CNAs) are part of the molecular makeup of breast cancer [19]. Multiple studies have reported an association between CNAs and specific tumor characteristics such as histologic grade, risk of recurrence, and metastasis [20–26]. CNAs have been reported to be of independent prognostic, even after adjustment for stage, histologic grade, TP53 status, histologic subtype and total aneuploidy [20].

In our previous large population based study, triple negative breast cancer (TNBC: ER negative, PR negative, absence of HER-2 overexpression) was the most frequently observed subtype in PABC compared to age-matched non-PABC tumors [27], in line with other case control studies [14, 18, 28, 29].

To assess whether this frequently observed subtype in PABC bears a unique molecular signature, we compared the genomic background of triple negative PABC and control non-pregnant breast cancer patients by detection of specific DNA copy number alterations. Associations between individual gene CNAs, clinicopathological characteristics and survival were explored.

## Materials and methods

### Patient selection

Using our Dutch nationwide population based ‘PABC cohort’ of women ≤ 45 years of age (*n* = 744), with a first diagnosis of invasive breast cancer (BC) during a first pregnancy or within six months after delivery [27], we extracted PABC patients with a triple negative receptor status (*n* = 283). Of these patients, breast tumor specimens have been requested from Dutch laboratories using the Dutch nationwide network and registry of histo- and cytopathology (PALGA) [30]. Only patients with full relevant clinical information about their outcome and available formalin-fixed paraffin-embedded material of their pregnancy associated breast tumor could be included for this molecular analysis (*n* = 31). As controls, triple negative and poorly differentiated tumors of 23 randomly selected premenopausal non-PABC patients (defined as first diagnosis of invasive BC without any sign of pregnancy in the patient history), were identified from the archives of the Department of Pathology at the University Medical Center Utrecht, The Netherlands.

All data from the PALGA database are pseudonymized by a trusted third party (ZorgTTP, Houten, The Netherlands). Consent was given by all Dutch laboratories for the storage of their data by PALGA, and for scientific use of these data. Use of anonymous or coded ‘left over’ material for scientific purposes does not require informed consent according to our institutional medical ethical review board and according to Dutch legislation [30–32].

### DNA extraction and multiplex ligation-dependent probe amplification

Hematoxylin–eosin stained slides were reviewed by an experienced pathologist (PJvD) to confirm and mark the presence of malignancy in tumor samples. Areas with lymphocytic infiltrate or ductal carcinoma in situ were avoided. The ratio of tumor cells compared to other cell types in the infiltrative tumor was determined and expressed as a percentage of the total number of cells. After deparaffinization in xylene, DNA was extracted from the marked tumor area on five 10-µm unstained sections. Areas were scraped off with a scalpel and specimens were heated at 90 °C for 15 min in 200 μL lysis buffer (lysis buffer: 50 mM Tris-HCI buffer, pH 8.0, 0.5% Tween 20). Then, 20 μL proteinase K solution (10 mg/ml; Roche, Almere, The Netherlands) was added, and the sample was incubated at 56 °C overnight (∼16 h) for lysis of the tissues. Inactivation of proteinase K was achieved by heating the sample for 15 min at 80 °C. The crude lysate was centrifuged for 10 min at 14,000 rpm, and 5 μL (50–100 ng) from the supernatant was used for each multiplex ligation-dependent probe amplification (MPLA) reaction according to the manufacturers’ instructions, using the P078-D2 breast tumor kit (MRC Holland, Amsterdam, The Netherlands) as before [33]. This probe mix contains 55 MLPA probes, including in total 41 probes for the following breast cancer associated chromosomal regions: 6q25 (*ESR1*), 7p11 (*EGFR*), 8p12-p11 (*ZNF703*, *FGFR1*, *ADAM9*, *IKBKB*), 8q13-q24 (*PRDM14*, *MTDH*, *MYC*), 11q13 (*CCND1*, *EMSY*), 16q22 (*CDH1*), 17q11-q25 (*CPD*, *MED1*, *ERBB2*, *CDC6*, *TOP2A*, *MAPT*, *PPM1D*, *BIRC5*), 19q12 (*CCNE1*) and 20q13 (*AURKA*). In addition, 14 reference probes are included which target copy number stable regions in various tumor types including breast cancer.

All tests were performed in duplicate on an ABI 9700 PCR machine (Applied Biosystems, Foster City, CA, USA). PCR products were analyzed on an ABI3730 capillary sequencer (Applied Biosystems). Gene copy numbers were analyzed using Genemapper (Applied Biosystems) and Coffalyser NET software (MRC-Holland). Six negative reference samples (two blood and four formalin-fixed paraffin embedded normal breast tissue specimens) were taken along in each MLPA run to normalize MLPA ratios. For genes with more than one probe present in the kit, the arithmetic mean of all the probe peaks of this gene in duplicate was calculated. A mean probe ratio value below 0.7 was defined as loss, a value between 0.7 and 1.3 was defined as normal, 1.3–2.0 as gain/low-level amplification, and values > 2.0 were defined as high-level amplification, as established previously [34].

### Statistics

CNA data was summarized and plotted using GraphPad Prism version 8.3.0 for Windows (GraphPad Software, San Diego, California USA). The web tool ClustVis was used to visualize CNA patterns and create heatmaps after unsupervised hierarchical cluster analysis using Ward’s linkage algorithm with Euclidean distance metrics [35].

Statistical analysis was performed using IBM SPSS statistics for Windows version 26.0.0.1. Differences in number of CNAs between PABC and non-PABC patients, and between PABC subgroups (clusters) were evaluated by independent samples t-test and ANOVA with post-hoc Tukey HSD test, respectively. Differences between categorical variables were examined by chi-square statistics or Fisher Exact test when indicated. Individual significance level was set at *p* < 0.05. Bonferroni-Holm Correction was applied for multiple comparisons. Overall survival curves were constructed using the Kaplan–Meier method and the log-rank test was used to test for significance. Multivariate survival analysis was done using a backward Cox proportional hazards model. Characteristics with a *p*-value < 0.10 in univariate analysis and potential confounders were included.

## Results

Table 1 compares the clinicopathologic characteristics of the selected PABC and non-PABC patients. All non-PABC and all but one PABC tumors were poorly differentiated (grade III) according to the modified Bloom-Richardson Scarff grading system [36]. Mean age of PABC and non-PABC patients was 33 (range 23 – 42) and 40 (range 29 – 48) years, respectively. The mean tumor percentage of the microscopic slides of PABC and non-PABC patients was 70.3% (SD ± 12.2%) and 70.9% (SD ± 11.6%) respectively, whilst the median tumor percentage was identical in both groups (70%, IQR 60–80%).

**Table 1:** Clinicopathologic characteristics of pregancy associated breast cancer (PABC) and non-PABC cohorts in this study

		PABC	non-PABC	
		*n* = 31	*n* = 23	significance
Age	**Range**	23–42	29–48	***P < 0,0001***
	**Mean**	33,3	40,3	
	**Stdev**	3,58	6,02	
cT	**1**	20,6	45,4	*p* = 0,110
*N* = *29 and 22*	**2**	58,6	40,9	
	**3**	17,2	13,6	
	**4**	3,4	0	
	**mm range (avg)**	15–100 (34)	11–75 (27)	
cN	**0**	79,3	71,4	*p* = 0,216
*N* = *29 and 21*	**1**	20,7	23,8	
	**2**	0	4,8	
	**3**	0	0	
cM	**0**	96,6	100	*p* = 1,000
*N* = *29 and 20*	**1**	3,4	0	
Surgery type	**MST + OKD**	24,1	39,1	***p = 0,029***
*N* = *29 and 23*	**MST—OKD**	44,8	8,7	
	**LMP + OKD**	13,8	13	
	**LMP—OKD**	17,2	39,1	
Mortality	**yes**	37,9	27,3	*p* = 0,424
*N* = *29 and 22*	**no**	62,1	72,7	
Follow-up (days)	**Range**	324–7367	420–5960	
	**Mean**	2744	3504	*p* = 0,106
	**Stdev**	2189	1703	
Stage	**1**	20	-	
*N* = *25*	**2**	64	-	
	**3**	12	-	
	**4**	4	-	
Trimester	**1**	17,9	-	
*N* = *28*	**2**	14,3	-	
	**3**	46,4	-	
	**postpartum**	21,4	-	

### TOP2A copy number loss is more frequent in triple negative PABC compared to non-PABC

**Fig. 1:**
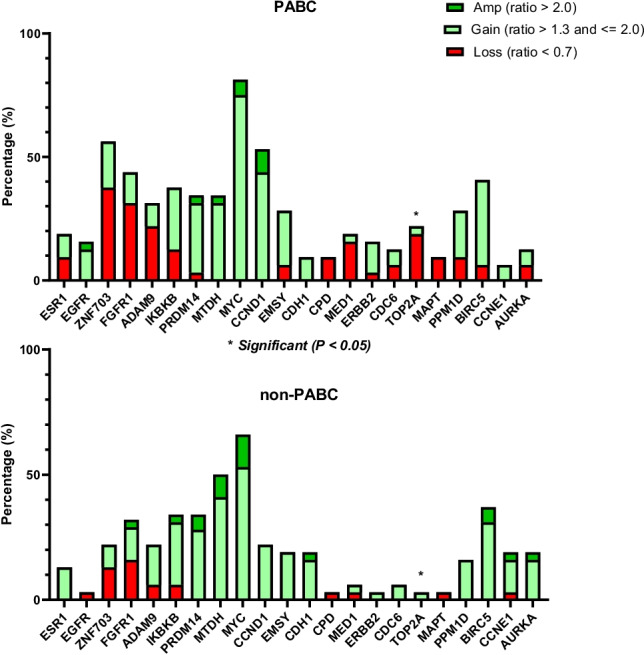
Copy number alteration (amplification, gain and loss) frequencies of 22 breast-cancer related genes in pregnancy associated breast cancer (PABC) and non-PABC

In general, PABC triple negative tumors showed significantly more losses (*p* = 0.046) and tended to show fewer high-level amplifications than non-PABC triple negative tumors. Table 2 compares the frequencies of individual gene CNAs between PABC and non-PABC cohorts, and shows mean and median MLPA copy number ratios per gene. *TOP2A* loss was frequent in PABC (19%) while it was not observed in non-PABC patients (*p* = 0.03; non-significant after correction for multiple comparisons). For all 21 other genes, no significant differences were observed. Figure 1 depicts observed frequencies of losses, gains and amplifications in PABC and non-PABC patients. *MYC* was the most frequently gained/amplified gene (81% and 66% of PABC and non-PABC patients, respectively). No *ESR1* and *ERBB2* (*HER2*) high-level amplifications were observed.

**Table 2:** Gene-specific frequencies of copy number alterations by MLPA in pregnancy-associated breast cancer (PABC) and non-PABC patients. Mean and median MLPA copy number ratio, including standard deviation (stdev) and interquartile range (iqr), as well as the results of inter-group statistical comparison, are also given

		PABC (*N* = 31)							non-PABC (*n* = 23)							chi-square statistics	
Gene	Chr	mean	stdev	median	iqr	loss	gain	HL amp	mean	stdev	median	iqr	loss	gain	HL amp	L vs N	GA vs N
ESR1	6q	0,99	0,26	0,92	0,28	9%	9%	0%	1,10	0,20	1,09	0,31	0%	13%	0%	0,25	0,69
EGFR	7p	1,12	0,32	1,05	0,19	0%	13%	3%	0,95	0,16	0,96	0,21	3%	0%	0%	0,46	0,13
ZNF703	8p	1,06	0,33	1,16	0,42	38%	19%	0%	1,07	0,25	1,05	0,22	13%	9%	0%	0,36	0,45
FGFR1	8p	1,04	0,29	1,04	0,33	31%	13%	0%	1,12	0,43	1,02	0,41	16%	13%	3%	0,77	1,00
ADAM9	8p	0,95	0,33	0,93	0,40	22%	9%	0%	1,04	0,27	1,00	0,39	6%	16%	0%	0,45	0,44
IKBKB	8p	1,10	0,25	1,07	0,27	13%	25%	0%	1,22	0,34	1,12	0,45	6%	25%	3%	1,00	0,54
PRDM14	8q	1,17	0,30	1,12	0,40	3%	28%	3%	1,37	0,45	1,21	0,29	0%	28%	6%	1,00	0,49
MTDH	8q	1,26	0,26	1,23	0,25	0%	31%	3%	1,46	0,36	1,42	0,45	0%	41%	9%	no loss	0,08
MYC	8q	1,57	0,34	1,63	0,51	0%	75%	6%	1,81	1,22	1,59	0,69	0%	53%	13%	no loss	0,98
CCND1	11q	1,44	0,65	1,27	0,46	0%	44%	9%	1,21	0,25	1,16	0,32	0%	22%	0%	no loss	0,27
EMSY	11q	1,04	0,24	0,98	0,23	6%	22%	0%	1,16	0,21	1,14	0,28	0%	19%	0%	0,50	0,87
CDH1	16q	1,08	0,17	1,09	0,21	0%	9%	0%	1,18	0,36	1,17	0,30	0%	16%	3%	no loss	0,26
CPD	17q	0,87	0,15	0,88	0,17	9%	0%	0%	0,93	0,12	0,96	0,16	3%	0%	0%	0,63	no GA
MED1	17q	0,94	0,22	0,91	0,27	16%	3%	0%	0,97	0,15	0,98	0,14	3%	3%	0%	0,23	1,00
ERBB2	17q	1,02	0,22	1,01	0,34	3%	13%	0%	0,99	0,18	0,95	0,16	0%	3%	0%	1,00	0,37
CDC6	17q	1,01	0,19	1,01	0,26	6%	6%	0%	1,08	0,23	1,03	0,17	0%	6%	0%	0,50	1,00
TOP2A	17q	0,93	0,22	0,92	0,31	19%	3%	0%	1,01	0,17	1,06	0,23	0%	3%	0%	**0,03**	1,00
MAPT	17q	0,92	0,18	0,92	0,28	9%	0%	0%	0,90	0,11	0,89	0,08	3%	0%	0%	1,00	no GA
PPM1D	17q	1,07	0,27	1,09	0,34	9%	19%	0%	1,07	0,27	0,96	0,22	0%	16%	0%	0,25	1,00
BIRC5	17q	1,15	0,26	1,16	0,34	6%	34%	0%	1,32	0,36	1,25	0,28	0%	31%	6%	0,51	0,51
CCNE1	19q	1,06	0,17	1,01	0,19	0%	6%	0%	1,16	0,35	1,08	0,20	3%	13%	3%	0,40	0,22
AURKA	20q	0,97	0,27	0,96	0,23	6%	6%	0%	1,22	0,57	1,15	0,34	0%	16%	3%	0,50	0,12

### Cluster analysis identifies triple negative PABC subgroup with poor outcome

**Fig. 2:**
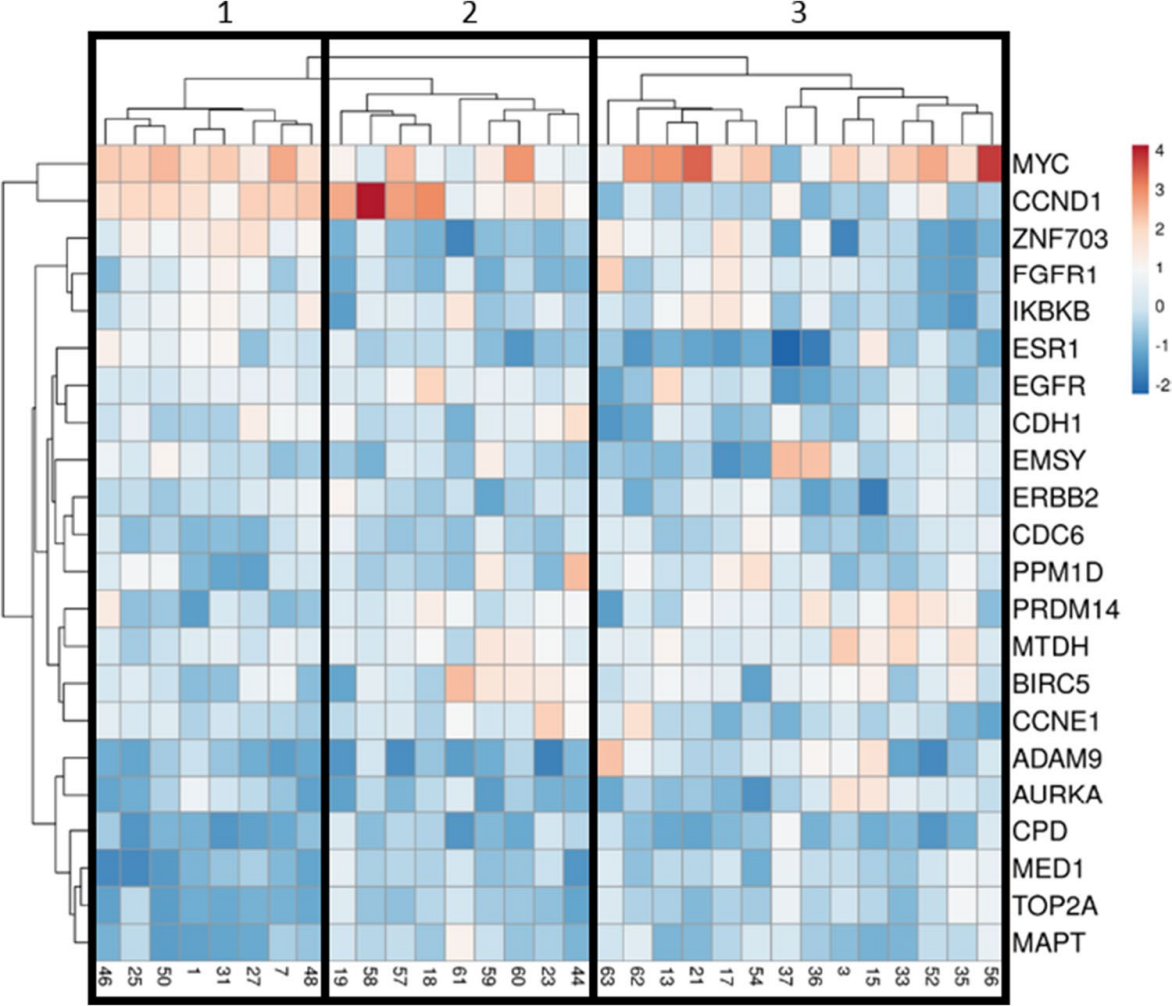
Unsupervised hierarchical cluster analysis of triple negative pregnancy associated breast cancer (PABC) patients based on somatic copy number alteration patterns of 22 breast cancer related genes. Cluster 2 was associated with significantly worse prognosis compared to cluster 1 and 3

**Fig. 3:**
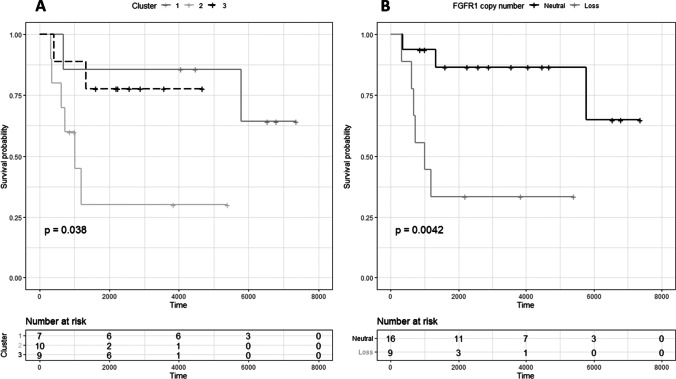
Kaplan Meier survival plots comparing outcome (**A**) in three pregnancy associated breast cancer (PABC) copy number alteration-classified subgroups identified by unsupervised hierarchical cluster analysis, and (**B**) patients with (ratio < 0.7) and without *FGFR1* copy number loss by multiplex ligation-dependent probe amplification

**Fig. 4:**
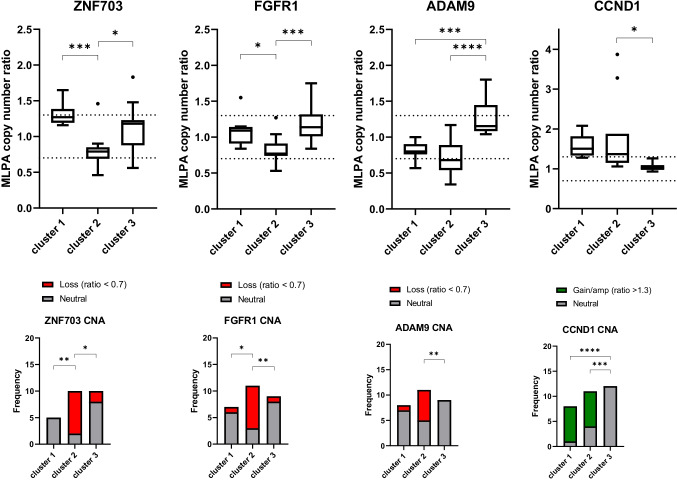
Differences in *ZNF703*, *FGFR1*, *ADAM9* and *CCND1* copy number between three triple negative pregnancy associated breast cancer (PABC) subgroups identified by unsupervised hierarchical cluster analysis (clusters 1, 2 and 3). Boxplots extend from the 25th to 75th percentiles. Whiskers and outliers were identified by the Tukey method. Cumulative number of patients with neutral copy number, loss and gain/amplification per cluster are shown in the bottom row. * *p* < 0.05; ** *p* > 0.01; *** *p* < 0.001

Unsupervised hierarchical cluster analysis of PABC and non-PABC patients based on CNA profiles revealed no clear distinction between both groups (Supplementary Fig. 1). Clustering within PABC patients, however, revealed 3 major clusters (Fig. 2) based on significant CNA differences between chromosomal regions 6q (*ESR1*), 8p (*ZNF703*, *FGFR1*, *ADAM9*), 11q (*CCND1*), 17q (*CPD*, *MED1*, *CDC6*, *TOP2A*, *MAPT*) and 20q (*AURKA*). Supplementary Table 1 provides an overview of the different clusters. One of these three clusters consisted of patients showing a far worse survival compared to the other triple negative PABC patients (*p* = 0.038; Fig. 3), and was characterized by more 8p loss (*ZNF703*, *FGFR1* and *ADAM9*) compared to the other two clusters (Fig. 4). No significant differences in gestational trimester, age at BC diagnosis or cTNM stage were observed between clusters.

### *FGFR1* and *TOP2A* copy number loss are independent prognosticators in triple negative PABC

CNAs individually associated with poor overall survival were *ESR1* loss (*n* = 3 events, *p* = 0.025), *FGFR1* loss (*n* = 9 evens, *p* = 0.0042; Fig. 3), *ADAM9* loss (*n* = 7 events, *p* = 0.037) and *CCNE1* gain (*n* = 2 events, *p* = 0.021). Patients presenting with tumors harboring *FGFR1* loss developed more frequently distant metastases (67% vs. 25% if copy number neutral, *p* = 0.048). Tumors harboring *MYC* gain or amplification were less likely to develop lymph node metastases (9% vs. 57% if copy number neutral, *p* = 0.018). *TOP2A* loss, *ESR1* loss, and *FGFR1* loss were independent predictors of overall survival (OS) in Cox regression analysis including cT and cN (HR 8.960 (95% CI 1.407–57.079), *p* = 0.020; HR 10.589 (95% CI 1.046–107.2108), *p* = 0.046; and HR 3.586 (95% CI 0.981–13.103), *p* = 0.053, respectively). Of these 3 CNAs, only *FGFR1* loss and *TOP2A* loss remained in the model when entered together (HR 4.408, *p* = 0.073 and HR 7.100, *p* = 0.056 respectively).

### Associations found in PABC are not seen in non-PABC

In the non-PABC group, no significant associations with survival were observed for *FGFR1* or any other interrogated gene, although *AURKA* gain/amp (*p* = 0.066) and *EMSY* gain/amp (*p* = 0.056) tended to predict worse survival. Unsupervised cluster analysis of non-PABC patients also revealed three clusters based on significant CNA differences between chromosomal regions 8p (ZNF703, FGFR1, ADAM9) and 17q (TOP2A, MAPT and BIRC5). All three patient clusters however had a similar survival (*p* = 0.463).

## Discussion

To investigate the underlying mechanisms resulting in the aggressive clinical behavior of PABC, we aimed to identify specific gene CNAs characterizing triple negative PABC, by conducting a comparative analysis of a cohort of triple negative PABC patients and subtype-matched non-PABC patients (with a diagnosis of invasive breast cancer ≤ 45 years of age). We have shown that triple negative PABC tumors exhibit enrichment for copy number losses by MLPA in general and some unique CNAs, including the enrichment for *TOP2A* copy number loss. In addition, *MYC* was the most frequently gained/amplified gene in PABC [37].

Cluster analysis based on CNA profiles identified a triple negative PABC subgroup with a particularly poor prognosis, characterized by chromosome 8p copy number loss. Further analysis of individual gene CNAs revealed that *FGFR1* copy number loss on chromosome 8p11.23 was the best prognosticator residing in this chromosomal region. *FGFR1* loss was an independent predictor of worse overall survival in multivariate analysis and predicted the development of distant metastases.

In line with our observations, other studies have previously described 8p copy number loss as a frequent event in various cancer types including breast cancer, suggesting that this region harbors one or more tumor suppressor genes. Loss of 8p has been linked to advanced tumor stage, high grade, high proliferation index, negative ER and PR status, early-onset breast cancer, poor survival rates and shortened response to oncologic systemic treatment [38–40]. Cai et al. examined the effect of a chromosome 8p 2–35 Mb targeted deletion, which was insufficient to transform MCF10A cells, but altered the fatty acid and ceramide metabolism leading to increased invasiveness and enhanced autophagy [41]. Their results provided evidence to suggest that screening for 8p loss in breast tumors may serve as a selection strategy for treatment with microtubule inhibitors (confers resistance), statins (confers resistance), and/or autophagy inhibitors (confers sensitivity). This strategy may thus be of particular interest in a PABC context.

Besides *FGFR1*, *TOP2A* copy number loss on 17q21.2, *ESR1* loss on chromosome 6q25.1 and *CCNE1* gain on 19q12 were identified as biomarkers for poor outcome in triple negative PABC patients. *TOP2A* loss, enriched in PABC tumors and covered by three independent MLPA probes, was an independent predictor of poor overall survival alongside *FGFR1* loss.

*TOP2A* encodes the topoisomerase IIα protein, an intracellular target of anthracyclines. Several studies have therefore suggested that anthracycline-containing therapy might be most effective in patients whose tumors carry amplified *TOP2A* [42–44]. Interestingly, *TOP2A* gene deletion might also confer increased sensitivity to anthracyclines [42, 45–47] suggesting a potential benefit of anthracycline-containing chemotherapy in triple negative PABC patients.

*ESR1* encodes the estrogen receptor alpha and, as expected since it usually leads to ER alpha overexpression, did not show amplifications in both triple negative cohorts. Yet, we did observe several *ESR1* losses in PABC tumors (9%; covered by two MLPA probes). Activating mutations in *ESR1* are recurrent mechanisms of acquired resistance to aromatase inhibitors in ER-positive tumors, but *ESR1* allelic losses have only rarely been described [48]. Laenkholm et al. reported that a large fraction of ER negative tumors showed *ESR1* deletion (55%) by FISH [49]. They also noted an elevated number of deletions in cohorts with a higher number of ER negative patients in the DBCG trial 89D. Thus *ESR1* deletions may contribute to the ER alpha negative status of these cancers.

Cyclin E1 (*CCNE1*) plays a critical role in cell cycle regulation, DNA replication, chromosome segregation, and G1 to S-phase transition [50]. CCNE1 overexpression and gene amplification have both been associated with poor prognosis in triple negative breast cancer [51–53] as well as epithelial ovarian cancer [54]. In ovarian cancer, the near mutual exclusivity of homologous recombination pathway mutations and *CCNE1* amplification generally results in resistance to platinum-based cytotoxic chemotherapies and ineffective Poly (Adenosine Diphosphate (ADP)-Ribose Polymerase (PARP) inhibition [54].

*CCNE1* amplified tumors can cause faster mitotic exit, an increased rate of mitotic slippage and resistance to anti-mitotic chemotherapies such as taxanes. Breast tumor cells engineered to overexpress cyclin E have been shown to have an increased sensitivity to cisplatin and paclitaxel combinations [55, 56]. Promising targeted strategies using CDK2 inhibitors and WEE1 kinase inhibitors are currently being examined in ongoing biomarker driven clinical trials.

The abovementioned prognostic CNAs proved to be unique to PABC tumors as similar associations were not observed in the breast tumors diagnosed outside pregnancy of postpartum period. This reinforces the notion that PABC represents an even more distinctive entity of breast cancer than previously reported, requiring its own biomarkers and therapeutic approaches.

Even though several studies on the genomic profile of PABC have been conducted [57], this analysis is novel as it focuses specifically on the triple-negative PABC subtype and correlates the clinicopathological features of the disease and outcomes with the CNAs. Although MLPA analysis alone cannot determine whether triple-negative PABC is defective in homologous recombination, recent genomic analysis has revealed that a significant portion of TNBC is characterized by abundant chromosomal structural variants and CNAs due to homologous recombination deficiency [58].

Some limitations of this study to be noted. Although MLPA is a multiplex technique that can assess multiple relevant CNAs simultaneously, we have only examined a limited number of genes here. PABC and non-PABC cohorts were relatively small but perfectly matched for triple negative subtype, and still provided prognostically significance. These new genetic insights can serve as a starting point for further more extensive copy number analyses by next generation sequencing in our entire PABC cohort, after obtaining the formalin-fixed paraffin-embedded (FFPE) tumor material of the remaining patients. In addition, age-matching was not perfect as PABC patients were on average slightly younger (33 years) than non-PABC patients (40 years) upon final analysis. Age was, however, not significantly associated with any of the interrogated variables, so we do not believe that this has played an important role here.

In conclusion, this study provides important new insights into the biology of triple negative PABC and suggests that several copy number alterations, particularly 8p loss, *TOP2A* loss, *ESR1* loss and *CCNE1* gain are implicated in tumor progression during pregnancy. *FGFR1* loss and *TOP2A* loss are promising new biomarkers that independently identify a subgroup of triple negative poor prognosis PABC patients that require personalized cancer treatment. In addition, this study provides unprecedented therapeutic clues for further studies to pursue in a larger PABC population.

## Supplementary Information

Below is the link to the electronic supplementary material.

Supplementary file1 (DOCX 69 kb)

## Data Availability

Research data on request.

## References

[1] Smith LH, Danielsen B, Allen ME, Cress R (2003). Cancer associated with obstetric delivery: results of linkage with the California cancer registry. Am J Obstet Gynecol..

[2] Andersson TM, Johansson ALV, Hsieh CC, Cnattingius S, Lambe M (2009). Increasing incidence of pregnancy-associated breast cancer in Sweden. Obstet Gynecol.

[3] Middleton LP, Amin M, Gwyn K, Theriault R, Sahin A (2003). Breast carcinoma in pregnant women: assessment of clinicopathologic and immunohistochemical features. Cancer.

[4] Amant F, Verheecke M, Wlodarska I, Dehaspe L, Brady P, Brison N, Van Den Bogaert K, Dierickx D, Vandecaveye V, Tousseyn T, Moerman P, Vanderstichele A, Vergote I, Neven P, Berteloot P, Putseys K, Danneels L, Vandenberghe P, Legius E, Vermeesch JR (2015). Presymptomatic Identification of Cancers in Pregnant Women During Noninvasive Prenatal Testing. JAMA Oncol..

[5] Lee GE, Mayer EL, Partridge A (2017). Prognosis of pregnancy-associated breast cancer. Breast Cancer Res Treat..

[6] Wang B, Yang Y, Jiang Z, Zhao J, Mao Y, Liu J, Zhang J (2019). Clinicopathological characteristics, diagnosis, and prognosis of pregnancy-associated breast cancer. Thorac Cancer..

[7] Zagouri F, Psaltopoulou T, Dimitrakakis C, Bartsch R, Dimopoulos MA (2013). Challenges in managing breast cancer during pregnancy. J Thorac Dis..

[8] Beadle BM, Woodward WA, Middleton LP, Tereffe W, Strom EA, Litton JK, Meric-Bernstam F, Theriault RL, Buchholz TA, Perkins GH (2009). The impact of pregnancy on breast cancer outcomes in women. Cancer.

[9] Rodriguez AO, Chew H, Cress R, Xing G, McElvy S, Danielsen B, Smith L (2008). Evidence of poorer survival in pregnancy-associated breast cancer. Obstet Gynecol..

[10] Bonnier P, Romain S, Dilhuydy JM, Bonichon F, Julien JP, Charpin C, Lejeune C, Martin PM, Piana L (1997). Influence of pregnancy on the outcome of breast cancer: a case-control study. Societe Francaise de Senologie et de Pathologie Mammaire Study Group. Int J Cancer..

[11] Shao C, Yu Z, Xiao J, Liu L, Hong F, Zhang Y, Jia H (2020). Prognosis of pregnancy-associated breast cancer: a meta-analysis. BMC Cancer.

[12] Johansson ALV, Andersson TM, Hsieh CC, Jirström K, Dickman P, Cnattingius S, Lambe M (2013). Stage at diagnosis and mortality in women with pregnancy-associated breast cancer (PABC). Breast Cancer Res Treat..

[13] Bae SY, Kim SJ, Lee J, Lee ES, Kim EK, Park HY, Suh YJ, Kim HK, You JY, Jung SP (2018). Clinical subtypes and prognosis of pregnancy-associated breast cancer: results from the Korean Breast Cancer Society Registry database. Breast Cancer Res Treat..

[14] Madaras L, Kovács KA, Szász AM, Kenessey I, Tőkés AM, Székely B, Baranyák Z, Kiss O, Dank M, Kulka J (2014). Clinicopathological features and prognosis of pregnancy associated breast cancer - a matched case control study. Pathol Oncol Res..

[15] Boudy AS, Naoura I, Selleret L, Zilberman S, Gligorov J, Richard S, Thomassin-Naggara I, Chabbert-Buffet N, Ballester M, Bendifallah S, Darai E (2018). Propensity score to evaluate prognosis in pregnancy-associated breast cancer: Analysis from a French cancer network. Breast.

[16] Amant F, von Minckwitz G, Han SN, Bontenbal M, Ring AE, Giermek J, Wildiers H, Fehm T, Linn SC, Schlehe B, Neven P, Westenend PJ, Müller V, Van Calsteren K, Rack B, Nekljudova V, Harbeck N, Untch M, Witteveen PO, Schwedler K, Thomssen C, Van Calster B, Loibl S (2013). Prognosis of women with primary breast cancer diagnosed during pregnancy: results from an international collaborative study. J Clin Oncol..

[17] Murphy CG, Mallam D, Stein S, Patil S, Howard J, Sklarin N, Hudis CA, Gemignani ML, Seidman AD (2012). Current or recent pregnancy is associated with adverse pathologic features but not impaired survival in early breast cancer. Cancer.

[18] Johansson ALV, Andersson TM, Hsieh CC, Jirström K, Cnattingius S, Fredriksson I, Dickman PW, Lambe M (2018). Tumor characteristics and prognosis in women with pregnancy-associated breast cancer. Int J Cancer..

[19] Futreal PA, Coin L, Marshall M, Down T, Hubbard T, Wooster R, Rahman N, Stratton MR (2004). A census of human cancer genes. Nat Rev Cancer..

[20] Zhang Y, Martens JW, Yu JX, Jiang J, Sieuwerts AM, Smid M, Klijn JG, Wang Y, Foekens JA (2009). Copy number alterations that predict metastatic capability of human breast cancer. Cancer Res..

[21] Smith JC, Sheltzer JM (2018). Systematic identification of mutations and copy number alterations associated with cancer patient prognosis. Elife.

[22] Hieronymus H, Schultz N, Gopalan A, Carver BS, Chang MT, Xiao Y, Heguy A, Huberman K, Bernstein M, Assel M, Murali R, Vickers A, Scardino PT, Sander C, Reuter V, Taylor BS, Sawyers SL (2014). Copy number alteration burden predicts prostate cancer relapse. Proc Natl Acad Sci U S A..

[23] Hirsch D, Kemmerling R, David S, Camps J, Meltzer PS, Ried T, Gaiser T (2013). Chromothripsis and focal copy number alterations determine poor outcome in malignant melanoma. Cancer Res..

[24] Han X, Tan Q, Yang S, Li J, Xu J, Hao X, Hu X, Xing P, Liu Y, Lin L, Gui L, Qin Y, Yang J, Liu P, Wang X, Dai W, Lin D, Lin H, Shi Y (2019). Comprehensive Profiling of Gene Copy Number Alterations Predicts Patient Prognosis in Resected Stages I-III Lung Adenocarcinoma. Front Oncol..

[25] Jones RA, Moorehead RA (2019). Integrative analysis of copy number and gene expression data identifies potential oncogenic drivers that promote mammary tumor recurrence. Genes Chromosomes Cancer..

[26] Pearlman A, Upudhyay K, Cole K, Loke J, Sun K, Fineberg S, Freedland SJ, Shao Y, Ostrer H (2018). Robust genomic copy number predictor of pan cancer metastasis. Genes Cancer..

[27] Suelmann BBM, van Dooijeweert C, van der Wall E, Linn S, van Diest PJ (2021). Pregnancy-associated breast cancer: nationwide Dutch study confirms a discriminatory aggressive histopathologic profile. Breast Cancer Res Treat..

[28] Asztalos S, Pham TN, Gann PH, Hayes MK, Deaton R, Wiley EL, Emmadi R, Kajdacsy-Balla A, Banerji N, McDonald W, Khan SA, Tonetti DA (2015). High incidence of triple negative breast cancers following pregnancy and an associated gene expression signature. Springerplus.

[29] Genin AS, Lesieur B, Gligorov J, Antoine M, Selleret L, Rouzier R (2012). Pregnancy-associated breast cancers: Do they differ from other breast cancers in young women?. The Breast..

[30] Casparie M, Tiebosch AT, Burger G, Blauwgeers H, van de Pol A, van Krieken JH, Meijer GA (2007). Pathology databanking and biobanking in The Netherlands, a central role for PALGA, the nationwide histopathology and cytopathology data network and archive. Cell Oncol..

[31] Dutch Government, Medical Research Involving Human Subjects Act, in Overheid Wettenbank The Hague, Rijksoverheid. http://www.ccmo-online.nl/main.asp?pid=10&sid=30&ssid=51 Accessed December 2021

[32] van Diest PJ (2002). No consent should be needed for using leftover body material for scientific purposes. For. BMJ..

[33] Moelans CB, de Weger RA, Monsuur HN, Vijzelaar R, van Diest PJ (2010). Molecular profiling of invasive breast cancer by multiplex ligation-dependent probe amplification-based copy number analysis of tumor suppressor and oncogenes. Mod. Pathol..

[34] Moelans CB, de Weger RA, van Diest PJ (2010). Multiplex ligation-dependent probe amplification to detect HER2 amplification in breast cancer: new insights in optimal cut-off value. Cell Oncol..

[35] Metsalu T, Vilo J (2015). Clustvis: a web tool for visualizing clustering of multivariate data using Principal Component Analysis and heatmap. Nucleic Acids Res..

[36] Bansal C, Singh US, Misra S, Sharma KL, Tiwari V, Srivastava AN (2012). Comparative evaluation of the modified Scarff-Bloom-Richardson grading system on breast carcinoma aspirates and histopathology. Cytojournal..

[37] Nguyen B, Venet D, Azim HA, Brown D, Desmedt C, Lambertini M, Majjaj S, Pruneri G, Peccatori F, Piccart M, Rothé F, Sotiriou C (2018). Breast cancer diagnosed during pregnancy is associated with enrichment of non-silent mutations, mismatch repair deficiency signature and mucin mutations. NPJ Breast Cancer..

[38] Lebok P, Mittenzwei A, Kluth M, Özden C, Taskin B, Hussein K, Möller K, Hartmann A, Lebeau A, Witzel I, Mahner S, Wölber L, Jänicke F, Geist S, Paluchowski P, Wilke C, Heilenkötter U, Simon R, Sauter G, Terracciano L, Krech R, von der Assen A, Müller V, Burandt E (2015). 8p deletion is strongly linked to poor prognosis in breast cancer. Cancer Biol Ther..

[39] Moelans CB, van Maldegem CMG, van der Wall E, van Diest PJ (2018). Copy number changes at 8p11-12 predict adverse clinical outcome and chemo- and radiotherapy response in breast cancer. Oncotarget.

[40] Armes JE, Hammet F, de Silva M, Ciciulla J, Ramus SJ, Soo WK, Mahoney A, Yarovaya N, Henderson MA, Gish K, Hutchins AM, Price GR, Venter DJ (2004). Candidate tumor-suppressor genes on chromosome arm 8p in early-onset and high-grade breast cancers. Oncogene.

[41] Cai Y, Crowther J, Pastor T, AbbasiAsbagh L, Baietti MF, De Troyer M, Vazquez I, Talebi A, Renzi F, Dehairs J, Swinnen JV, Sablin AA (2016). Loss of Chromosome 8p Governs Tumor Progression and Drug Response by Altering Lipid Metabolism. Cancer Cell..

[42] Di Leo A, Gancberg D, Larsimont D, Tanner M, Jarvinen T, Rouas G, Dolci S, Leroy JY, Paesmans M, Isola J, Piccart MJ (2002). HER-2 amplification and topoisomerase IIalpha gene aberrations as predictive markers in node-positive breast cancer patients randomly treated either with and anthracycline-based therapy or with cyclophosphamide, methotrexate, and 5-fluorouracil. Clin Cancer Research..

[43] Slamon D, Eiermann W, Robert N, Pienkowski T, Martin M, Rolski J, Chan A, Mackey J, Liu M, Pinter T, Valero V, Falkson C, Fornander T, Shiftan T, Olsen S, Buyse M, Kiskartalyi T, Landreau V, Wilson V, Press MF, Crown J (2009). Phase III Randomized Trial Comparing Doxorubicin and Cyclophosphamide Followed by Docetaxel (AC→T) with Doxorubicin and Cyclophosphamide Followed by Docetaxel and Trastuzumab (AC→TH) with Docetaxel, Carboplatin and Trastuzumab (TCH) in Her2neu Positive Early Breast Cancer Patients: BCIRG 006 Study. Cancer Res..

[44] Press MF, Sauter G, Buyse M, Bernstein L, Guzman R, Santiago A, Villalobos IE, Eiermann W, Pienkowski T, Martin M, Robert N, Crown J, Bee V, Taupin H, Flom KJ, Tabah-Fisch I, Pauletti G, Lindsay MA, Riva A, Slamon DJ (2011). Alteration of topoisomerase II-alpha gene in human breast cancer: association with responsiveness to anthracycline-based chemotherapy. J Clin Oncol..

[45] Knoop AS, Knudsen H, Balslev E, Rasmussen BB, Overgaard J, Nielsen KV, Schonau A, Gunnarsdóttir K, Olsen KE, Mouridsen H, Ejlertsen B (2005). Danish Breast Cancer Cooperative Group, retrospective analysis of topoisomerase IIa amplifications and deletions as predictive markers in primary breast cancer patients randomly assigned to cyclophosphamide, methotrexate, and fluorouracil or cyclophosphamide, epirubicin, and fluorouracil: Danish Breast Cancer Cooperative Group. J Clin Oncol..

[46] O’Malley FP, Chia S, Tu D, Shepherd LE, Levine MN, Bramwell VH, Andrulis IL, Pritchard KI (2009). Topoisomerase II alpha and responsiveness of breast cancer to adjuvant chemotherapy. J Natl Cancer Inst..

[47] Bartlett JM, McConkey CC, Munro AF, Desmedt C, Dunn JA, Larsimont DP, O'Malley FP, Cameron DA, Earl HM, Poole CJ, Shepherd LE, Cardoso F, Jensen MB, Caldas C, Twelves CJ, Rea DW, Ejlertsen B, Di Leo A, Pritchard KI (2015). Predicting Anthracycline Benefit: TOP2A and CEP17-Not Only but Also. J Clin Oncol..

[48] Dustin D, Gu G, Fuqua SAW (2019). ESR1 mutations in breast cancer. Cancer.

[49] Laenkholm AV, Knoop A, Ejlertsen B, Rudbeck T, Jensen MB, Müller S, Lykkesfeldt AE, Rasmussen BB, Nielsen KV (2012). ESR1 gene status correlates with estrogen receptor protein levels measured by ligand binding assay and immunohistochemistry. Mol Oncol..

[50] Bendris N, Lemmers B, Blanchard JM (2015). Cell cycle, cytoskeleton dynamics and beyond: the many functions of cyclins and CDK inhibitors. Cell Cycle.

[51] Zhao ZM, Yost SE, Hutchinson KE, Li SM, Yuan YC, Noorbakhsh J, Liu Z, Warden C, Johnson RM, Wu X, Chuang JH, Yuan Y (2019). CCNE1 amplification is associated with poor prognosis in patients with triple negative breast cancer. BMC Cancer.

[52] Huang X, Shao D, Wu H, Zhu C, Guo D, Zhou Y, Chen C, Lin Y, Lu T, Zhao B, Wang C, Sun Q (2020). Genomic Profiling Comparison of Germline BRCA and Non-BRCA Carriers Reveals CCNE1 Amplification as a Risk Factor for Non-BRCA Carriers in Patients With Triple-Negative Breast Cancer. Front Oncol.

[53] Yuan Q, Zheng L, Liao Y, Wu G (2021). Overexpression of CCNE1 confers a poorer prognosis in triple-negative breast cancer identified by bioinformatic analysis. World J Surg Oncol..

[54] Gorski JW, Ueland FR, Kolesar JM (2020). CCNE1 Amplification as a Predictive Biomarker of Chemotherapy Resistance in Epithelial Ovarian Cancer. Diagnostics (Basel)..

[55] Smith ML, Seo YR (2000). Sensitivity of cyclin E-overexpressing cells to cisplatin/taxol combinations. Anticancer Res..

[56] Hunt KK, Keyomarsi K (2005). Cyclin E as a prognostic and predictive marker in breast cancer. Semin Cancer Biol..

[57] Korakiti AM, Moutafi M, Zografos E, Dimopoulos MA, Zagouri F (2020). The Genomic Profile of Pregnancy-Associated Breast Cancer: A Systematic Review. Front Oncol..

[58] Belli C, Duso BA, Ferraro E, Curigliano G (2019). Homologous recombination deficiency in triple negative breast cancer. Breast.

