# Supplementary material for: Genomic copy number alterations as biomarkers for triple negative pregnancy-associated breast cancer
Source: Cell Oncol (Dordr). 2022 Jul 6;45(4):591–600. doi: 10.1007/s13402-022-00685-6 (PMC9424154; doi:10.1007/s13402-022-00685-6)

**Supplementary Table 1.** Differences between three triple negative pregnancy associated breast cancer (PABC) subgroups identified by unsupervised hierarchical cluster analysis of somatic copy number alterations determined by multiplex ligation-dependent probe amplification (clusters 1, 2 and 3). Differences in absolute MLPA copy number ratio were determined by ANOVA with Tukey posthoc test. Differences in CNA frequency between clusters were determined by Chi square test. NS = non-significant

|  |  | **MLPA copy number ratio differences** | | | | **CNA frequency differences** | | | |
| --- | --- | --- | --- | --- | --- | --- | --- | --- | --- |
| ***Gene*** | ***chr arm*** | ***1 vs 2*** | ***1 vs 3*** | ***2 vs 3*** | ***direction*** | ***1 vs 2*** | ***1 vs 3*** | ***2 vs 3*** | ***direction*** |
| ***ESR1*** | **6q** | 0.018 | NS | NS | 1>2 | NS | NS | NS | - |
| ***ZNF703*** | **8p** | 0.001 | NS | 0.021 | 1/3>2 | 0.007 | NS | 0.023 | loss 2>1/3 |
| ***FGFR1*** | **8p** | 0.039 | NS | 0.001 | 1/3>2 | 0.028 | NS | 0.005 | loss 2>1/3 |
| ***ADAM9*** | **8p** | NS | 0.0003 | 0.000006 | 3>1/2 | NS | NS | 0.006 | loss 2>3 |
| ***CCND1*** | **11q** | NS | NS | 0.015 | 2>3 | NS | 0.0001 | 0.001 | GA 1/2>3 |
| ***CPD*** | **17q** | NS | 0.002 | 0.019 | 3>1/2 | NS | NS | NS | - |
| ***MED1*** | **17q** | NS | 0.00005 | 0.003 | 3>1/2 | NS | NS | NS | - |
| ***CDC6*** | **17q** | NS | NS | 0.017 | 3>2 | NS | NS | NS | - |
| ***TOP2A*** | **17q** | NS | 0.00004 | 0.001 | 3>1/2 | NS | NS | NS | - |
| ***MAPT*** | **17q** | NS | 0.001 | 0.013 | 3>1/2 | NS | NS | NS | - |
| ***AURKA*** | **22q** | NS | NS | 0.024 | 3>2 | NS | NS | NS | - |

**Supplementary Figure 1.** Unsupervised hierarchical cluster analysis of pregnancy associated breast cancer (PABC; blue) and non-PABC patients (red) based on somatic copy number alterations of 22 breast cancer related genes.


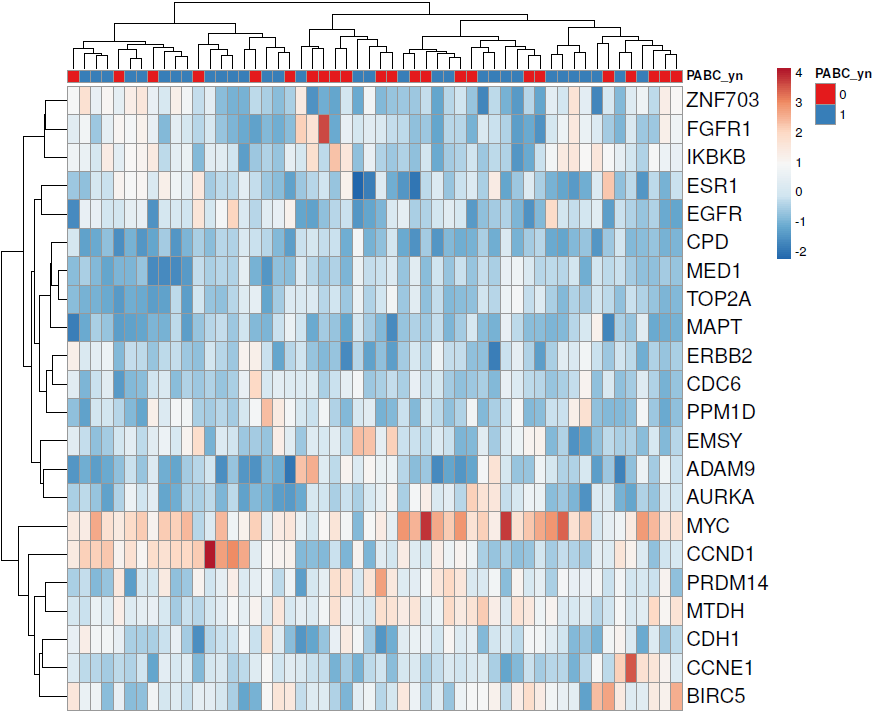

Supplement: Supplementary file 1 — Supplementary file1 (DOCX 69 kb) [file 13402_2022_685_MOESM1_ESM.docx]
